# Graphene coated dielectric resonator antenna for modeling the photoreceptors at visible spectrum

**DOI:** 10.1016/j.heliyon.2022.e09611

**Published:** 2022-06-01

**Authors:** Mahdi NoroozOliaei, Hamid Riazi Esfahani, Mohammad Sadegh Abrishamian

**Affiliations:** a Electrical & Computer Engineering Department, K. N. Toosi University of Technology, Tehran, Iran; b Farabi Eye Hospital, Tehran University of Medical Science, Tehran, Iran

**Keywords:** Dielectric resonator antenna, Electromagnetic model, Graphene, Photoreceptor, Retina

## Abstract

The absorption of light is very important task for retina photoreceptors. Graphene is an energy harvesting material and one of the best models for the electromagnetic wave absorption and its conversion into signals. In this paper, an electromagnetic modeling of human retinal photoreceptors has been presented based on graphene coated material as a receiver antenna. The proposed electromagnetic model based on dielectric resonator antenna (DRA) is being analyzed for retina photoreceptors of human eye (cones and rods) by Finite Integral Method (FIM) collaborated with CST MWS. The results show that the model is good for vision spectrum with a proper field enhancement in cone photoreceptor due to its sensitivity to light. The results indicate proper S_11_ (return loss below −10 dB) with invaluable resonances in a wide range of frequencies from 405 THz to 790 THz (vision spectrum), suitable S_21_ (insertion loss 3-dB bandwidth), very good field distribution for flowing the power within desired radiation characteristics. The drawbacks of conventional model (no coating) have been resolved by presenting this one at blue spectrum specifically. Finally, mfERG clinical and experimental results show that this model can stimulate the electrochemical voltages and currents in photoreceptor cells.

## 1 Introduction

Electromagnetic modeling of retina photoreceptors is a vital procedure in order to replace a prosthesis as a bionic eye. In recent years, blind people are searching for a proper device to restore their sights. That is why one of the electromagnetic challenges is analysis and modeling of retina and its optic nerve in order to replace a prosthesis as a bionic eye. There have been many good endeavors for realizing commercial prosthesis such as Argus I, Argus II (the second sight), Alpha-IMS, fractal prosthesis (FracRet AIM), and etc. [1], [2], [3], [4], [5], [6], [7].

Actually, there is no existing model (fabricated as mass production) as the view point of antenna concept. The graphene coated model is a unique and novel model. This is the first time that photoreceptors model is being introduced as a viewpoint of antenna concept.

**Figure 1:**
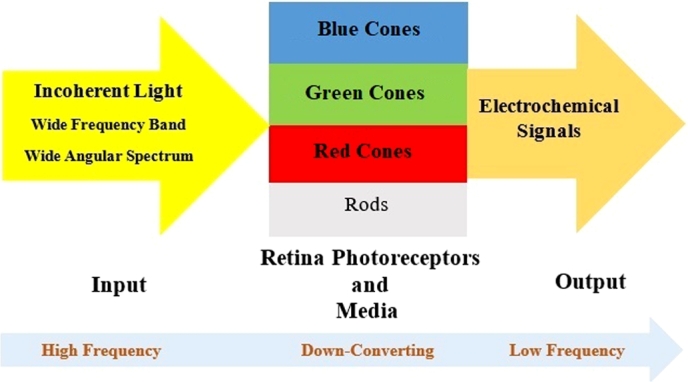
Block Diagram of Retina Photoreceptor System.

The incoming light waves are converted to electrical signals that stimulate neurons in retina and then convey information to other structures in the central nervous system (CNS) by optic nerve. The typical human trichromatic color visual system combines the response of three types of cone cells in retina; the three cone types-labeled for short, medium, and long wavelengths are sensitive primarily to light with central wavelengths of 437 nm (Blue), 533 nm (Green), and 610 nm (Red), respectively. The rod cells are also receiving the signal with a center wavelength of 498 nm for lower luminance of light. A block diagram of retina photoreceptor system has been shown in Fig. 1. The input is the incoherent light in visible spectrum.

In this paper, the proposed electromagnetic model on graphene coated dielectric resonator antenna (DRA) is being analyzed for retina photoreceptors of human eye (within its interior medium) by Finite Integral Method (FIM) collaborated with CST MWS. A comparison is done between two models i.e., graphene coated and no coating ones.

## 2 Modeling of photoreceptor

**Figure 2:**
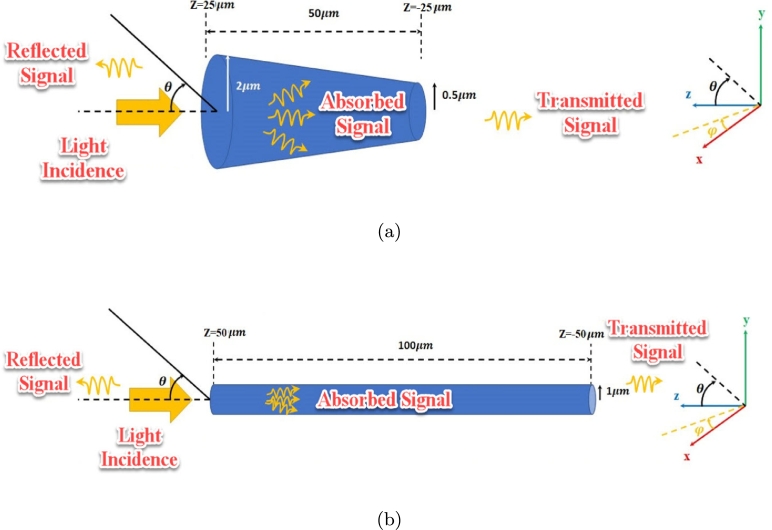
Light incidence to (a) conical and (b) cylindrical resonators.

The human tissue can act like either an absorber or a reflector at high frequencies; Blocking, attenuating, or redirecting any signal that propagates in its direction. Model of two photoreceptors is illustrated in Fig. 2.

The conical resonator is a chromatic one, three types of pigment (R, G, and B); it dominates in photopic vision by more than 3 cd/m^2^ and is responsible for different wavelength detection (color detection) but the rods are achromatic, one type of pigment; Rods dominate in scotopic vision by less than 0.003 cd/m^2^ and are responsible for intensity detection.

A human rod cell is about 2 microns in diameter and 100 microns long and the cone cells are typically 40-50 μm long, and their diameter varies from 0.5 to 4 μm [8].

## 3 Graphene and simulation environment

Computer Simulation Technology (CST) MWS is a special tool for the 3D electromagnetic simulation of high frequency components. It enables the fast and accurate analysis. In this broadband structure, it helps to simulate accurately.

**Figure 3:**
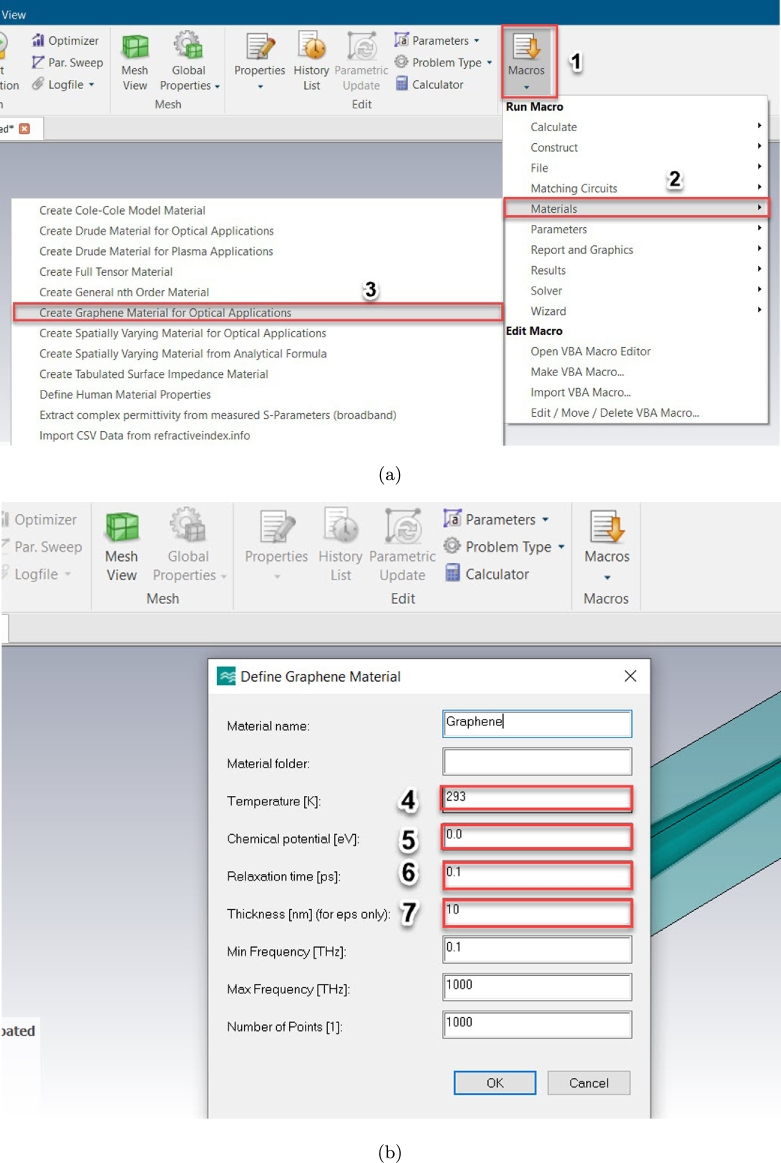
Definition of graphene material in CST MWS environment (a) first and (b) final procedures.

There are several stages for defining the graphene material in CST MWS as shown in Fig. 3:

1- Click on Macros (Top right button).

2- Click on Materials.

3- Choose the “Create Graphene Material for Optical Applications” Option.

4- Enter the temperature in K.

5- Enter the chemical potential in eV.

6- Enter the carrier relaxation time in ps.

7- Enter the thickness of graphene material already created in the CST environment.

The graphene is single layer of graphite. A planar monoatomic layer of carbon bonded in a hexagonal structure.

The Kubo model for the surface conductivity of graphene material can be shown as following equation [9]:

(1) $$\sigma_{g}\left(\omega ,\mu_{c},\Gamma ,T\right)=\frac{-je^{2}k_{B}T}{\pi \hbar^{2}\left(\omega -j2\Gamma \right)}\left[\frac{\mu_{c}}{k_{B}T}+2ln\left(e^{\frac{-\mu_{c}}{k_{B}T}}+1\right)\right]-\frac{je^{2}}{4\pi \hbar }ln\left(\frac{2\left|\mu_{c}\right|-\left(\omega -j2\Gamma \right)\hbar }{2\left|\mu_{c}\right|+\left(\omega -j2\Gamma \right)\hbar }\right)$$

Where *ω* is radian frequency, $\mu_{c}$ is the chemical potential or Fermi level which can be controlled by an applied electrostatic bias field $E_{0}$ or by doping, Γ is a phenomenological electron scattering rate (inverse of the carrier relaxation time of *τ*) that is assumed to be independent of energy, and *T* is temperature.

**Figure 4:**
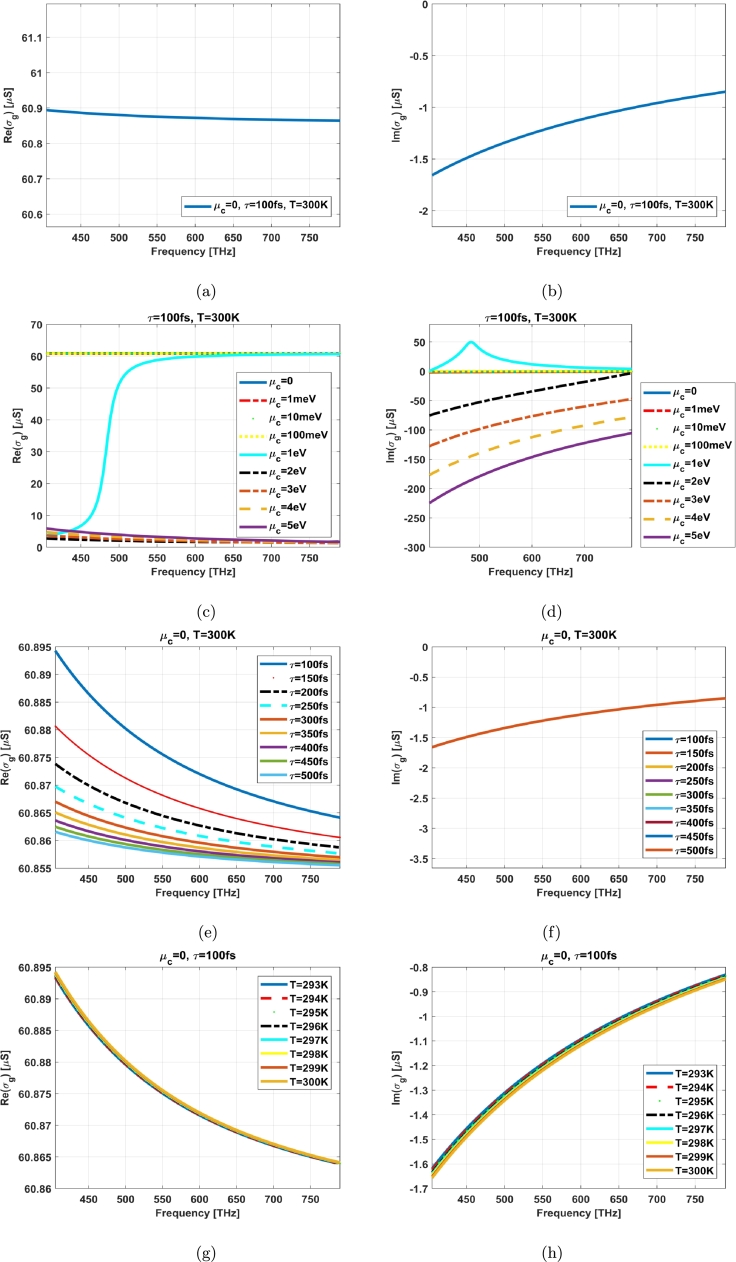
Real part for surface conductivity of graphene in visible spectrum with specified conditions: (a), (c), (e), (g), Imaginary part for surface conductivity of graphene in visible spectrum with specified conditions: (b), (d), (f), (h).

A code using MATLAB software has been terminated in accordance with the equation (1) in order to illustrate the real and imaginary parts of graphene surface conductivity in the vision spectrum (405 THz to 790 THz). Fig. 4 (a) and (b) show the real and imaginary parts of graphene surface conductivity by $\mu_{c}=0$, τ=100fs, T=300K.

An equivalent effective permittivity for the graphene as a very thin metal can be defined as following [10], [11]:

(2) εeffg(ω)=ε0+jσg(ω)ω

**Figure 5:**
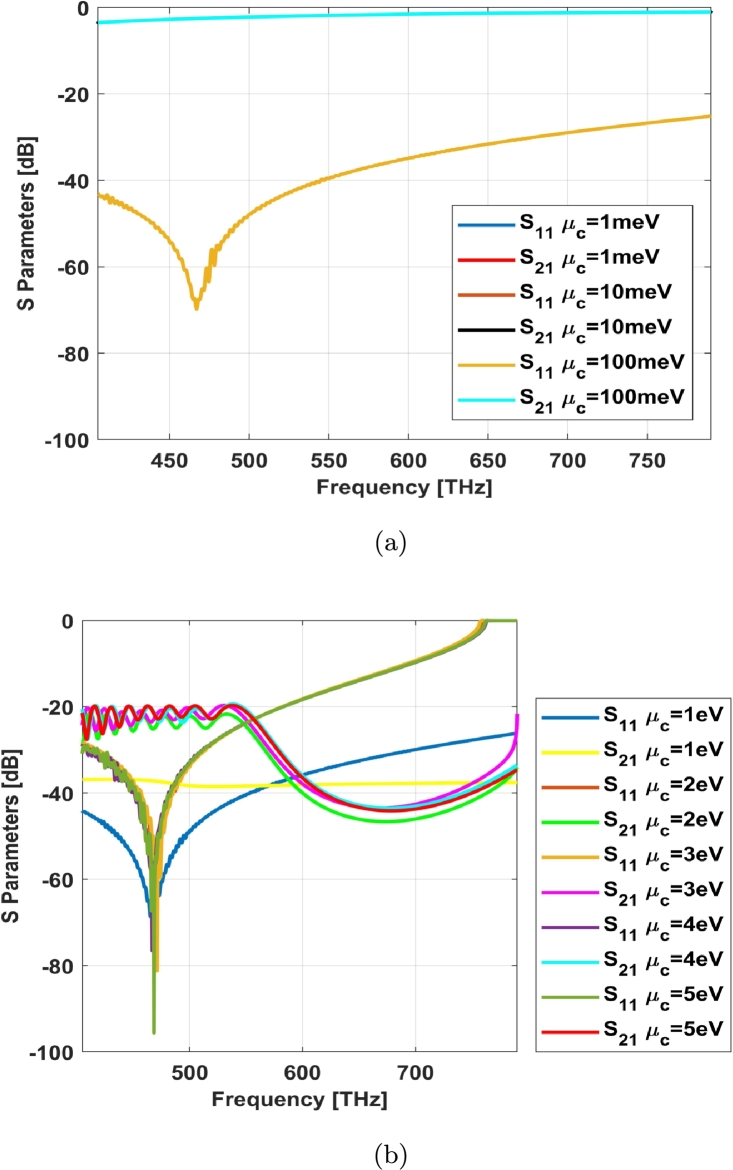
The effect of changing the chemical potentials on the simulated S parameters of proposed model (*τ* = 100 fs, *T* = 300 K): (a) low potentials and (b) 1 eV to 5 eV.

It means that real part of effective permittivity for graphene corresponds to imaginary part of its surface conductivity and vice versa regarding to the equation (2). The graphene behaves like a thin metal with a plasma frequency depending on Fermi level. As it can be seen from Fig. 4, the most effective parameter on graphene material at visible spectrum (405 THz to 790 THz) is the chemical potential. Fig. 5 shows the effects of chemical potential on the proposed model; it behaves very good at low chemical potentials due to the near zero epsilon property. It is noteworthy that the graphene in proposed model is not in a planar form (it can be estimated planar in small area), but also it is an enclosed layer over the photoreceptor. So, the inserted chemical potential over graphene layer impacts on the transverse fields (radial fields) and longitudinal (propagating) ones simultaneously. As the propagation constants of those fields are related, then increment in one side (for example transversal fields) cause decrement in other side (for example longitudinal fields).

In other words, the structure (port) is supported by Hybrid Mode (HEM), both TE and TM. Hybrid electromagnetic modes (HEM) are supported by structures exhibiting certain boundary condition(s) satisfying which requires a hybrid of TE and TM mode solutions, rather than either. These modes are usually found as the higher order modes of a waveguide or cavity structure, possessing some unique characteristics. Two structures that use the hybrid modes for popular but distinct applications are the (i) Iris loaded cylindrical waveguide and the (ii) Cylindrical dielectric resonator. Usually, hybrid mode structures are operated in the dominant hybrid mode designated as the HEM_11_ for waveguides and HEM_11*δ*_ for cavities [12], [13]. The chemical potential applies some effects to the fields in the direction of k_transversal_ (wavenumber in transversal directions) in addition to the k_longitudinal_ (wavenumber in longitudinal direction) due to its radial shape around the media and photoreceptor (dielectric part). It generates an extra applied electric field mostly in the radial (transversal) direction. This field impacts on both transversal and longitudinal fields simultaneously as they are related to each other for supporting the HEM mode.

The carrier relaxation time of electrons is estimated to be in the specified range of Fig. 4 (e) and (f) with respect to the change in complex permittivity in accordance with the Dirac massless fermions in graphene. On the other hand, the carrier relaxation time variation doesn't affect on surface conductivity substantially as shown in Fig. 4 (e) and (f). The room temperature in kelvin starts from 293K and it reaches to near 300K in a hot day. The ambient temperature also doesn't affect basically in accordance with Fig. 4 (g) and (h). The imaginary part of graphene surface conductivity (real part of epsilon) has a near zero value when the chemical potential is in the range of 0 to 1 eV (same values). It will be raised to a positive number at 1 eV and then returned to the negative one with a greater slope (Fig. 4(d)). Generally, the nonlinearity property on graphene surface conductivity specially with the specified condition in Fig. 4 (a) and (b) concludes to better results in transmitted signal as illustrated in Fig. 5.

When $Im\left(\sigma_{g}\right)$ is negative (Fig. 4 (b),(d),(f),(h)), graphene can also support the TE forms of Surface Plasmons (SPs) modes [14], [15]. This model has the potential for application to graphene-based plasmonic devices in photonics and optoelectronics, such as sensors in preparing the prosthesis device and bionic eye.

## 4 Simulation and results

**Figure 6:**
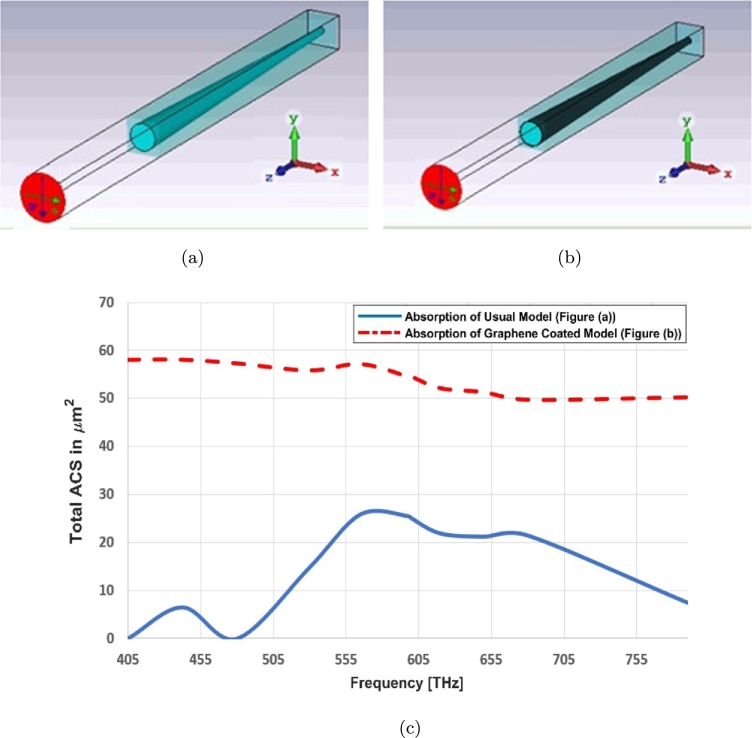
(a) Simple (usual) model (b) graphene coated model, (c) absorption cross section of (a) and (b).

The incidence procedure is defined by absorbed, transmitted and reflected signals as shown in Fig. 2. As an example, the absorption (total ACS) of simple (no coating) and graphene coated photoreceptor models are investigated as shown in Fig. 6. Several publications are indicating the optical index of refraction (square root of the dielectric constant) of the human eye and its photoreceptors [16], [17], [18].

It indicates that the absorption of graphene coated photoreceptor is roughly acceptable in all spectrum (Fig. 8 shows the absorption index more accurately).

**Figure 7:**
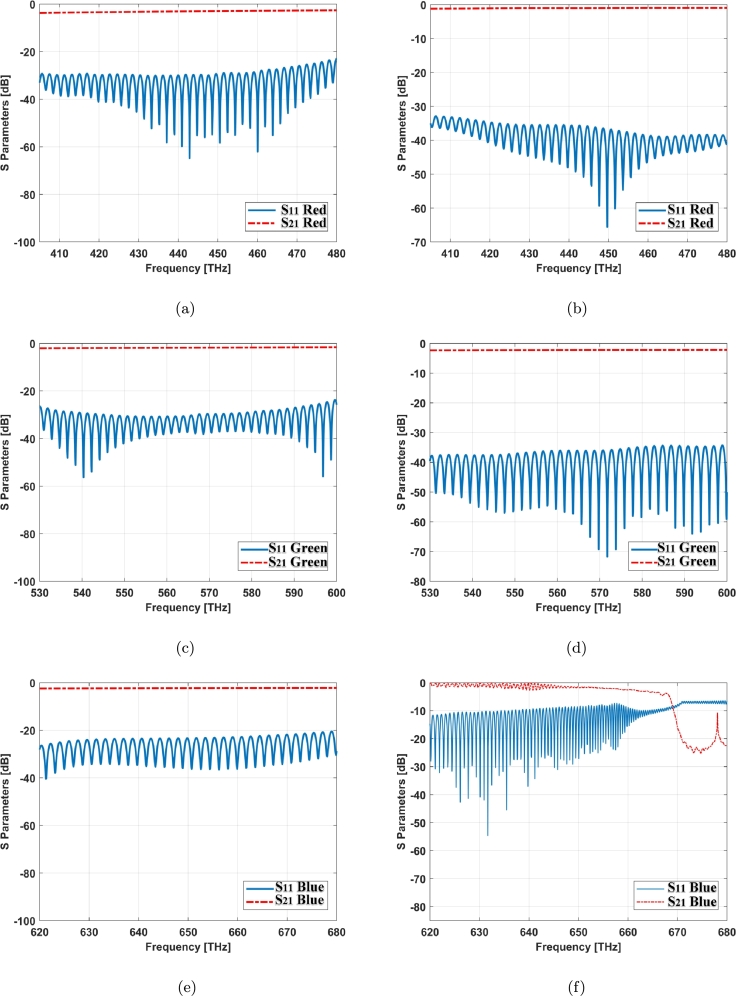
S Parameters for cone cell of graphene coated model: (a) red light, (c) green light, (e) blue light and simple model (no coating): (b) red light, (d) green light, (f) blue light.

**Figure 8:**
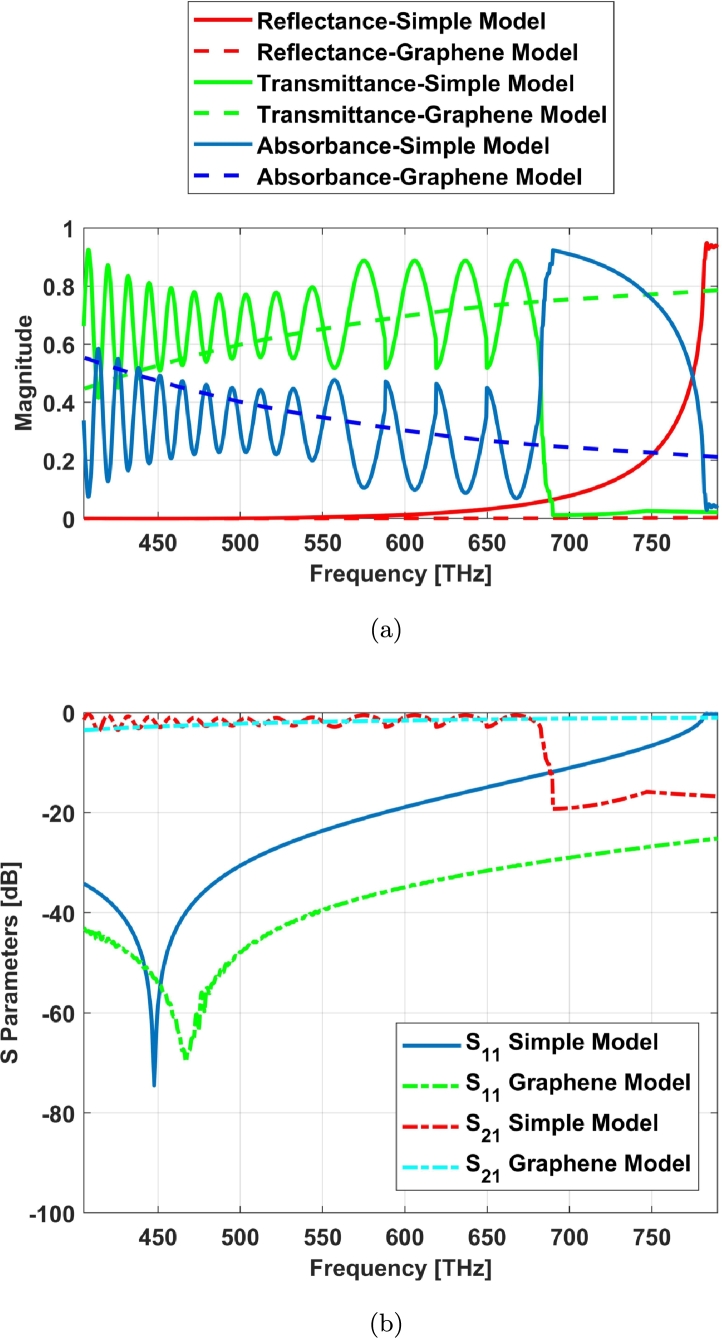
Simple and graphene coated cone photoreceptor models (a) S parameters and (b) absorbance, reflectance and transmittance indexes.

The nonlinearity effect of graphene surface conductivity and its near zero epsilon property help to decrease the reflection index and improve the transmittance profile (Figure 7, Figure 8). This helps for improving the field distribution in this structure.

The trichromatic colors i.e. red, green and blue lights are being transmitted separately to these two cone cell models as shown in Fig. 7. Fig. 7 (a), (c), and (e) show the S parameters of graphene coated cone model for these trichromatic colors while Fig. 7 (b), (d), and (f) illustrate the S parameters of simple cone model or no coating. Regarding to this result, the S parameters have been improved in blue light (Fig. 7 (e) and (f)).

Graphene tunes the reflection and transmission parameters of the structures based on the dispersive property (Fig. 8). The resonant frequencies can be dynamically modulated by changing the geometry and adjusting the graphene chemical potential [19].

Graphene-based plasmonic crystals present linear, elliptical, or hyperbolic dispersion relations that exhibit epsilon near zero (ENZ) behavior, normal or negative-index diffraction. The optical properties can be dynamically tuned by controlling the operating frequency and the doping level of graphene [20].

The real part of epsilon for graphene at visible spectrum (shown in Fig. 4) corresponds to the scale of micro siemens and it is too small (near zero). Combining with the dielectric photoreceptor and media (DRA) makes a behavior like graphene epsilon near zero plasmonic crystal.

The wide spectrum response, transmitted, reflected and absorbed signals of graphene coated model has been illustrated in Fig. 8. In the graphene coated model, absorption in red region is better than blue one and the transmittance factor has the opposite relationship with the absorption in this case.

**Figure 9:**
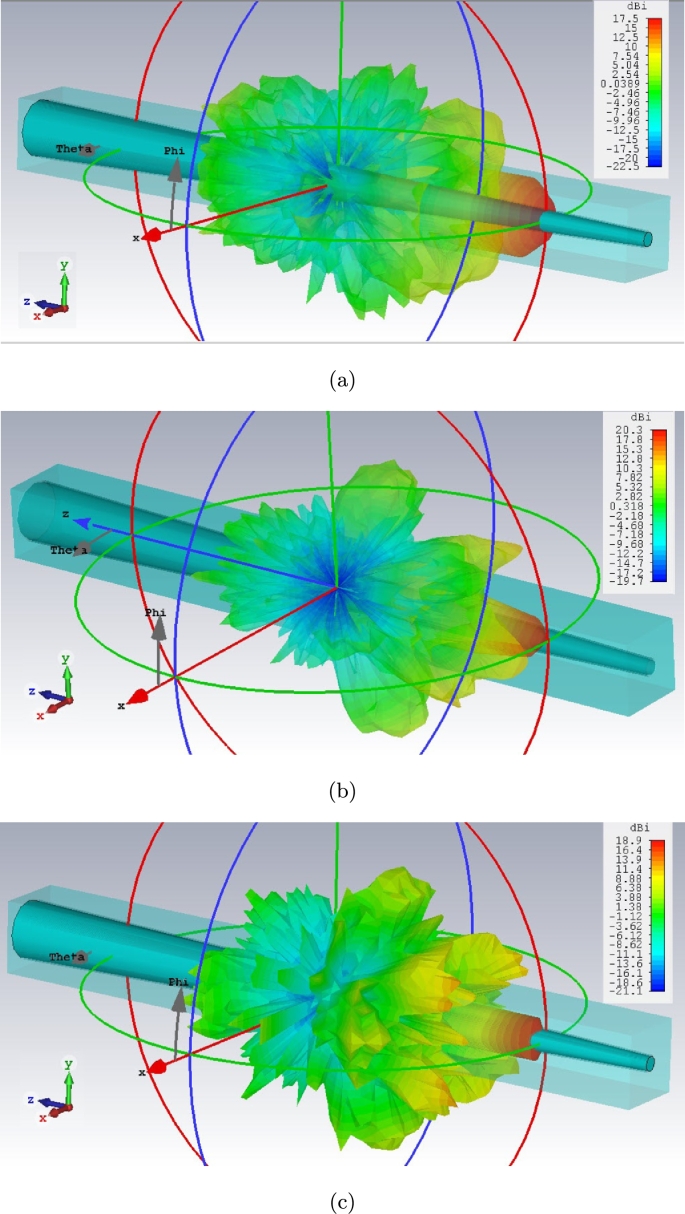
Far field radiation characteristics for cone cell of graphene coated model in red, green, and blue lights spectrum: (a) 442.5 THz, (b) 565 THz, and (c) 650 THz.

Fig. 9 expresses the proper directivity of this cone antenna model in all spectrum with no back-lobe direction.

**Figure 10:**
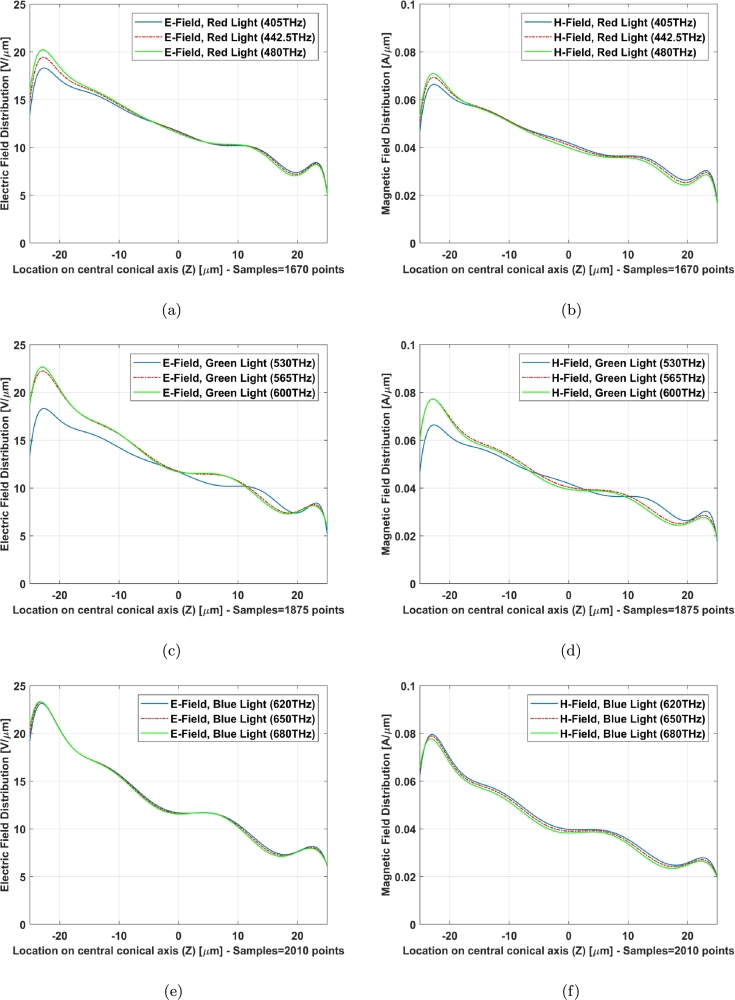
Graphene coated model of cone cell on Z axis (from Z = 25 μm to Z = −25 μm), E-field distribution: (a) red light, (c) green light, (e) blue light; H-field distribution: (b) red light, (d) green light, (f) blue light.

**Figure 11:**
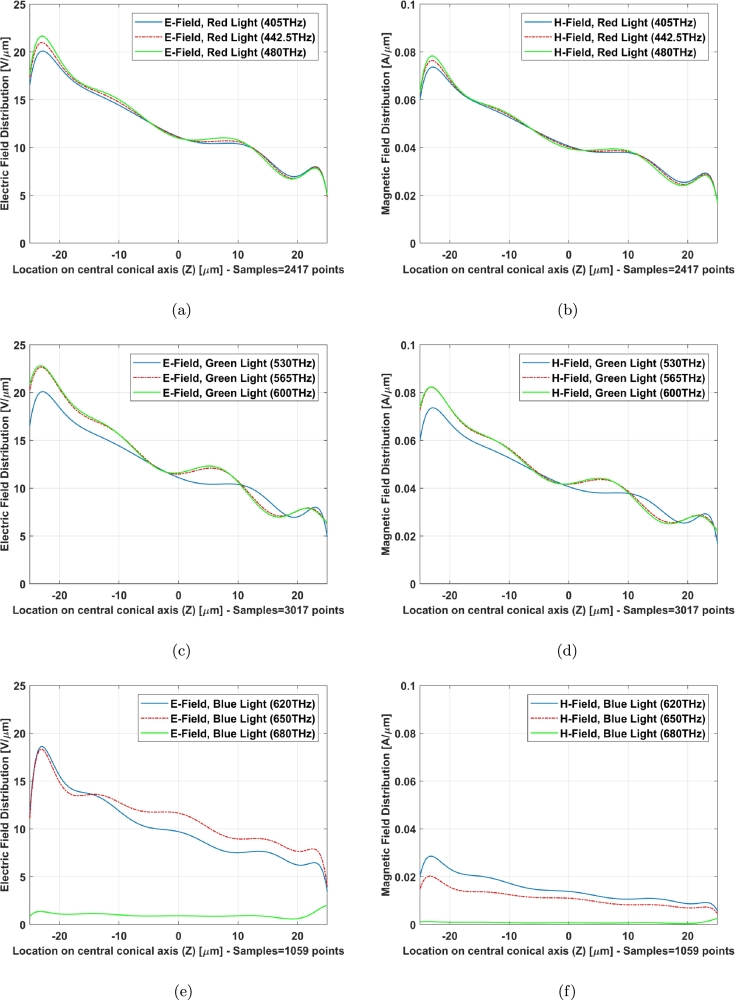
Simple model of cone cell on Z axis (from Z = 25 μm to Z = −25 μm), E-field distribution: (a) red light, (c) green light, (e) blue light; H-field distribution: (b) red light, (d) green light, (f) blue light.

The field distribution (Electric and Magnetic) of two models along Z axis (X=0, Y=0), central axis of structure as an example, indicates a familiar phenomenon nominated localized field enhancement [21] specially in cone photoreceptors (Figure 10, Figure 11). Figure 10, Figure 11 illustrate E-field and H-field distributions of graphene coated and simple models of cone photoreceptor, respectively. Fig. 10 (a), (c), and (e) indicate the electric field distribution of graphene coated cone model at red, green and blue regions meanwhile this parameter has been shown for simple cone model in Fig. 11 (a), (c), and (e). The magnetic field distribution of graphene coated cone model has been depicted in Fig. 10 (b), (d), and (f) at red, green and blue regions while Fig. 11 (b), (d), and (f) illustrate this parameter for simple cone model or no coating.

The field distribution in graphene coated model has gotten an improvement with respect to the simple one specially at higher frequencies of blue light (Fig. 10 (e) and (f) can be compared with Fig. 11 (e) and (f)).

**Figure 12:**
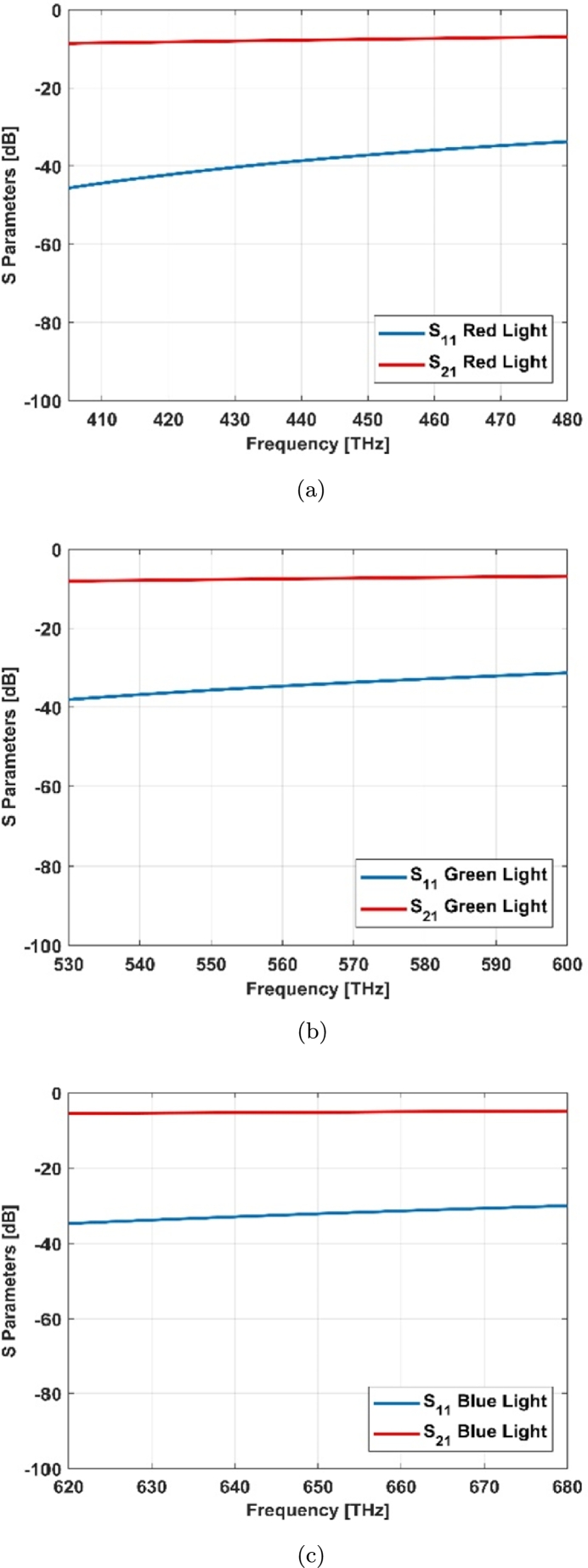
S Parameters for rod cell of graphene coated model: (a) red light, (b) green light, (c) blue light.

Some parameters of graphene coated cylindrical resonator (rod cell) are shown in the following. Fig. 12 depicts the S parameters for red, green, and blue spectrums, correspondingly.

**Figure 13:**
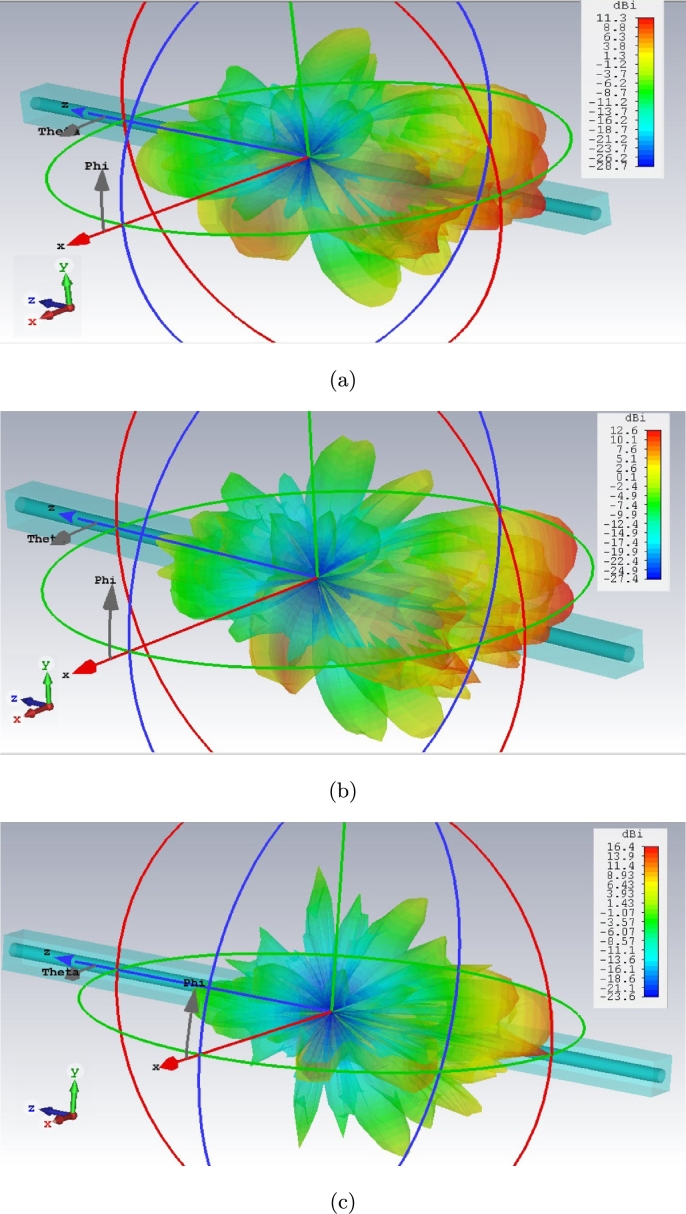
Far field radiation characteristics for rod cell of graphene coated model in red, green, and blue lights spectrum: (a) 442.5 THz, (b) 565 THz, and (c) 650 THz.

The far-field characteristics of graphene coated rod cell are being illustrated in Fig. 13.

**Figure 14:**
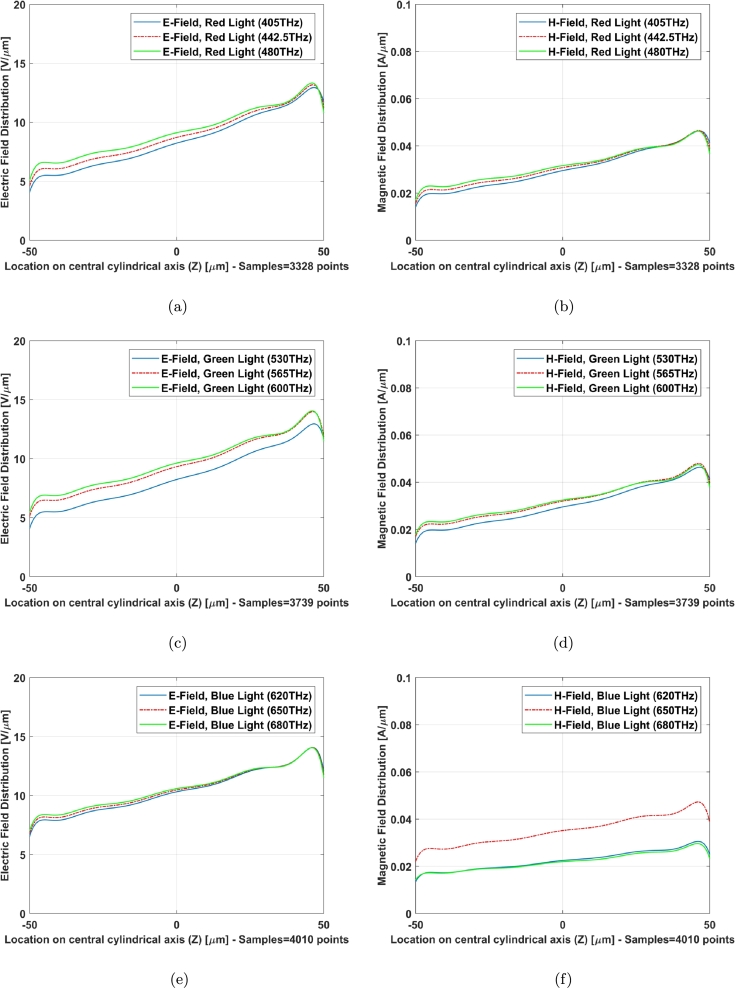
Graphene coated model of rod cell on Z axis (from Z = 50 μm to Z = −50 μm), E-field distribution: (a) red light, (c) green light, (e) blue light; H-field distribution: (b) red light, (d) green light, (f) blue light.

Fig. 14 shows that there is no significant field enhancement for rod cell, but the level has been retained in its good value due to graphene coated material as rod cell receives lower luminance of signals in scotopic vision. In other words, it requires less light to function than cone.

**Figure 15:**
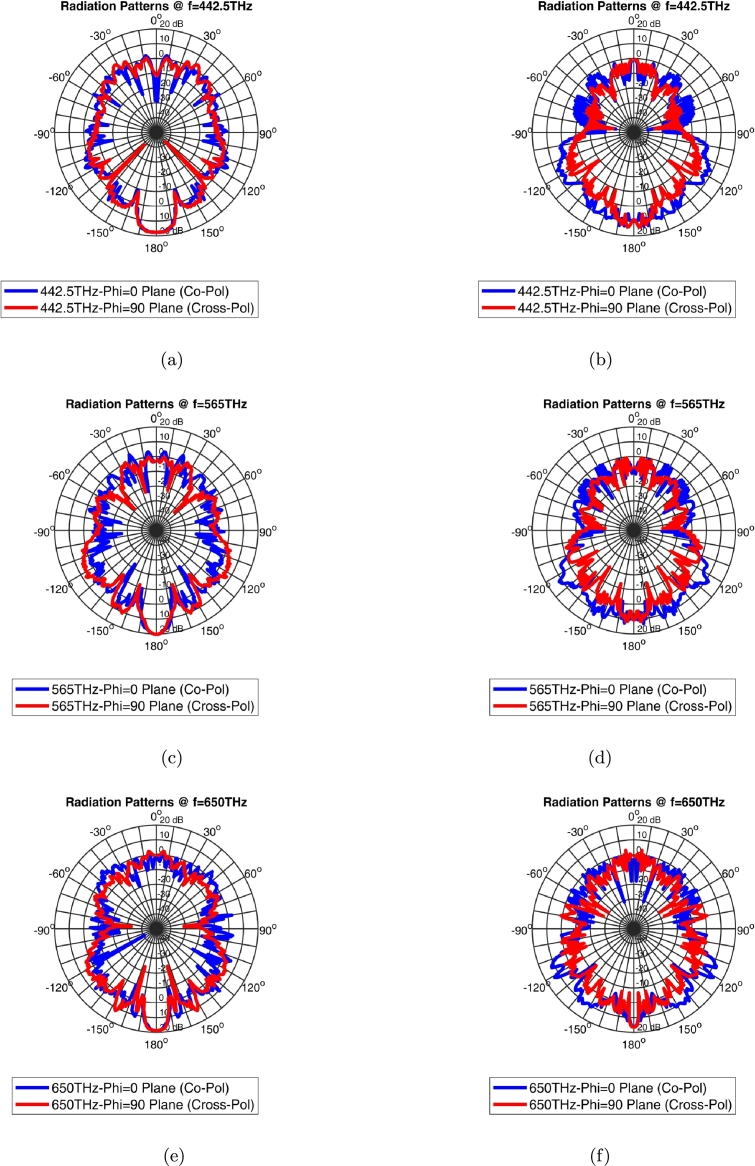
Co-polarization and Cross-polarization of single graphene coated cone and rod models (a) cone - 442.5 THz, (b) rod - 442.5 THz, (c) cone - 565 THz, (d) rod - 565 THz, (e) cone - 650 THz, (f) rod - 650 THz (0° and 180° are the angles alongside the Z axis and photoreceptor length-0°: to the light incidence side and 180°: to the end of photoreceptor).

The Co-polarization and Cross-polarization of graphene coated cone and rod models have been illustrated in Fig. 15 at 442.5 THz, 565 THz, and 650 THz.

**Figure 16:**
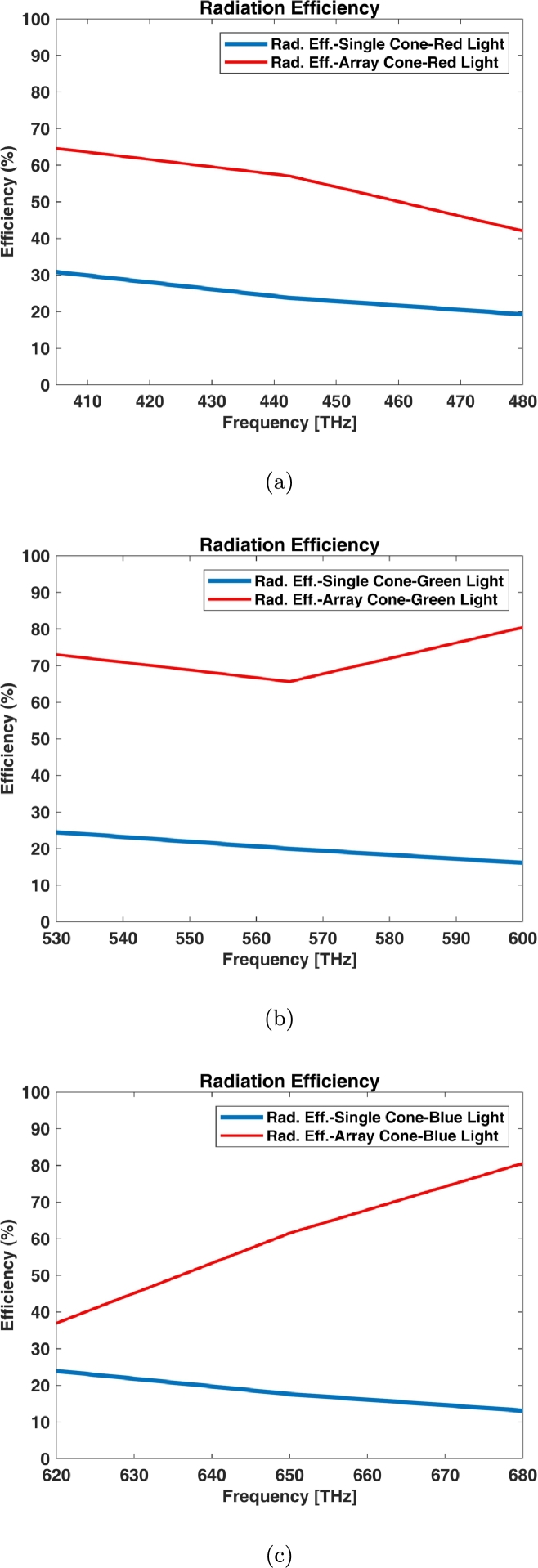
Radiation efficiency of single and array (3 × 3) for graphene coated cone models: (a) Red Light, (b) Green Light, and (c) Blue Light.

Radiation efficiency of a single graphene coated model is not significant as its array. This parameter has been compared for both single and array (3×3) in Fig. 16.

Directivity is a fundamental antenna parameter that shows a measure of how well the antenna directs the received energy toward a particular direction. As a receiver antenna, the directivity is more important than the gain. This is a model for the photoreceptors of retina in a human eye. So, it is a receptor not a transmitter. The gain will be meaningful when the antenna transmits the signal. This model indicates that how much values of the received energy will reach to the end of photoreceptors as electrochemical signals; then it defines the directivity parameter not the gain.

The gain of antenna can be related to directivity as the following equation [22]:

(3) $$G=\xi_{R}D$$

Which *G*, $\xi_{R}$ and *D* are gain, radiation (antenna) efficiency and directivity of antenna respectively. The gain can be realized by the mentioned directivity and the radiation efficiency (Figure 15, Figure 16) in accordance with equation (3).

The antenna has been analyzed in the viewpoint of receiver antenna. So, the investigation of the antenna gain and radiation efficiency doesn't matter for this case; But these types of antennas have a significant directivity to deliver the received power to a certain direction (This criterion is very important for realizing the artificial or bionic eye as the natural eye in retina layers and photoreceptors which direct the converted received photon energy to the electrochemical signals). The radiation efficiency of single graphene coated antenna is not so much (between 10-25 percent) at visible frequencies; but its array (for example 3×3) has a different story (near 50 to 80 percent radiation efficiency) as shown in Figure 15, Figure 16.

## 5 Experimental and clinical results

**Figure 17:**
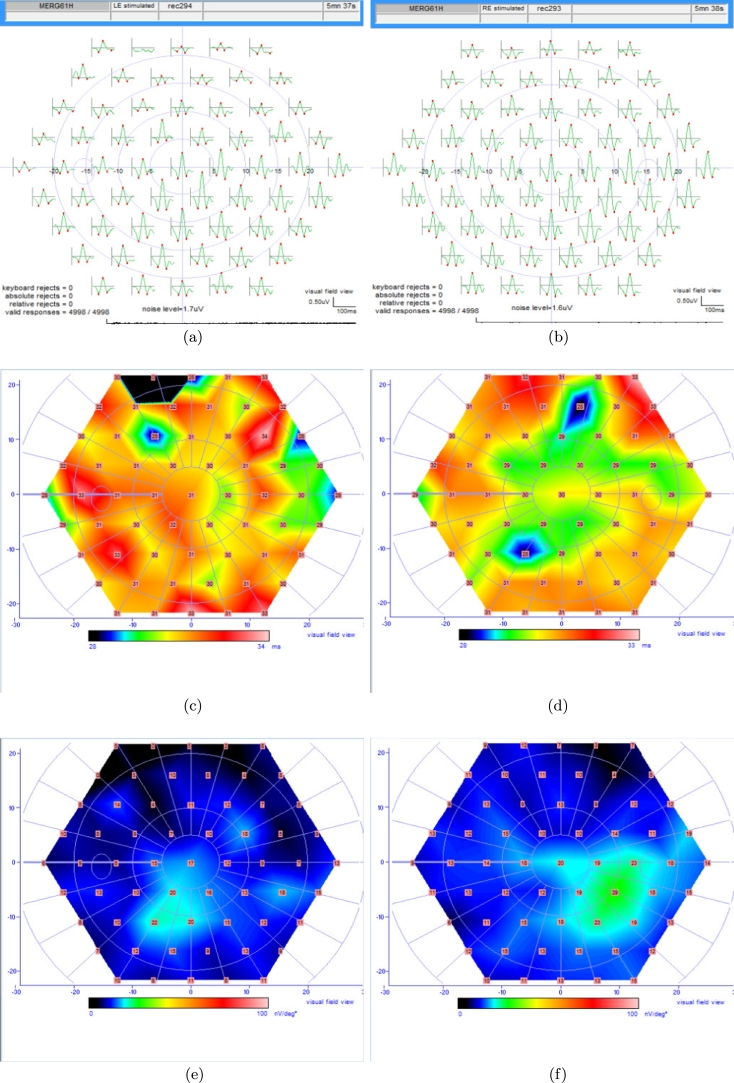
Left Eye of a Normal Subject (a) Field Trace Array, (c) N1 Implicit Time, (e) N1 Amplitude; Right Eye of a Normal Subject (b) Field Trace Array, (d) N1 Implicit Time, (f) N1 Amplitude.

The multifocal ERG usually is recorded for cone photoreceptors primarily in photopic state. mfERG responses are presented in the form of a scaled hexagonal pattern includes local ERG responses as shown in Figs. 17 (c)-(f).

The maximum light induced electrical response (voltage variable) of retinal cells is the amplitude. The mfERG waveform includes three points N1, the first negative peak, P1, the positive peak, and N2, the second negative peak. The overall mfERG amplitude is defined from N1 to P1. It shows the largest amplitude at the fovea region. The amplitude of the N1 is the average voltage of the encompassed window for N1 point; the N1 amplitude is from the baseline to the N1 point.

Implicit time (the time to peak) is the required time for the electrical response to reach the maximum amplitude. It is measured from stimulation time to the corresponding wave-component peak one and returns the signal conduction rate.

The array of traces in the top row (Figs. 17 (a) and (b)) illustrates the mfERG response obtained from the hexagons [23], [24].

It is noteworthy that N1 wave includes contributions from photoreceptors in comparison to other cells such as bipolars that contribute to the P1 wave [25], [26].

## 6 Discussion and analysis

The electromagnetic model based on dielectric resonator antenna (DRA) has been analyzed for retina photoreceptors of human eye (cones and rods) by Finite Integral Method (FIM) collaborated with CST MWS. The results show that the model is good for vision spectrum with a proper field enhancement in cone photoreceptor due to its sensitivity to light. The results indicate proper S_11_ (return loss below -10 dB) with invaluable resonances in a wide range of frequencies from 405 THz to 790 THz (vision spectrum), suitable S_21_ (insertion loss 3-dB bandwidth), very good field distribution for flowing the power within desired radiation characteristics.

The comparison of two models, graphene coated and simple one shows that there are significant improvements specially in blue light and beyond of spectrum. It can be expressed that many drawbacks of previous model have been covered by this graphene coated model.

The received level signal (field distributions) of the proposed model regarding to the input level can stimulate the signals of retina photoreceptors in accordance with the amplitude of clinical results obtained by mfERG technique (Table 1 in [25]).

This work focuses explicitly on the optical nerves of the human eye (photoreceptors). However human visual system contains other parts beyond optics that may be a good reference for future works. In [27], the ventral post-processing has been formulated in a robust and fast way actually. A complete eye model would involve all stages.

## 7 Conclusion

In this paper, an electromagnetic modeling of human retinal photoreceptors was presented based on graphene coated material as an antenna concept. The proposed electromagnetic model based on dielectric resonator antenna (DRA) was analyzed for retina photoreceptors of human eye (cones and rods) by Finite Integral Method (FIM) collaborated with CST MWS. The results showed that the model was good for vision spectrum with a proper field enhancement in cone photoreceptor due to its sensitivity to light. The results indicated proper S_11_ (return loss below -10 dB) with invaluable resonances in a wide range of frequencies from 405 THz to 790 THz (vision spectrum), suitable S_21_ (insertion loss 3-dB bandwidth), very good field distribution for flowing the power within desired radiation characteristics. Finally, clinical and experimental technique, mfERG, was compared to the numeric electromagnetic fields.

## Declarations

### Author contribution statement

Mahdi NoroozOliaei, Hamid Riazi Esfahani, Mohammad Abrishamian: Conceived and designed the experiments; Performed the experiments; Analyzed and interpreted the data; Contributed reagents, materials, analysis tools or data; Wrote the paper.

### Funding statement

This research did not receive any specific grant from funding agencies in the public, commercial, or not-for-profit sectors.

### Data availability statement

Data included in article/supp. material/referenced in article.

### Declaration of interest's statement

The authors declare no conflict of interest.

### Additional information

No additional information is available for this paper.

## Figures and Tables

**Figure:**
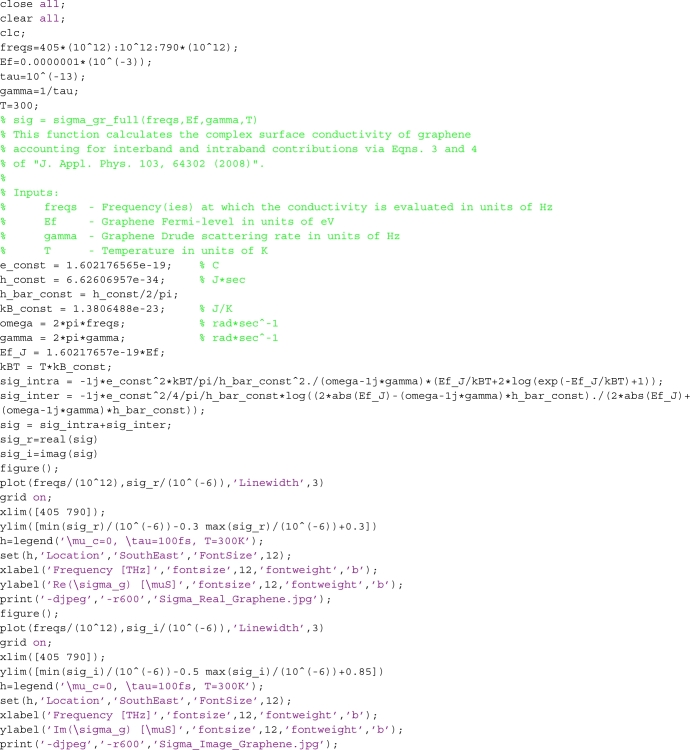


## Appendix A

The algorithms of MATLAB code have been added here:
